# Can we use local climate zones for predicting malaria prevalence across sub-Saharan African cities?

**DOI:** 10.1088/1748-9326/abc996

**Published:** 2020-12-15

**Authors:** O Brousse, S Georganos, M Demuzere, S Dujardin, M Lennert, C Linard, R W Snow, W Thiery, N P M van Lipzig

**Affiliations:** 1 Department of Earth and Environmental Sciences, KU Leuven, Leuven, Belgium; 2 UCL Institute for Environmental Design and Engineering, University College London, London, United Kingdom; 3 Department of Geosciences, Environment and Society, Université Libre de Bruxelles, Brussels, Belgium; 4 Department of Geography, Ruhr-University Bochum, Bochum, Germany; 5 Department of Environment, Ghent University, Ghent, Belgium; 6 Department of Geography, Université de Namur, Namur, Belgium; 7 Population and Health Unit, Kenya Medical Research Institute Wellcome Trust, Nairobi, Kenya; 8 Department of Tropical Medicine and Global Health, Nuffield Department of Medicine, University of Oxford, Oxford, United Kingdom; 9 Department of Hydrology and Hydraulic Engineering, Vrije Universiteit Brussel, Brussels, Belgium

**Keywords:** malaria, sub-Saharan africa, local climate zones, urban malaria modeling, random forest modeling, urban health, WUDAPT

## Abstract

Malaria burden is increasing in sub-Saharan cities because of rapid and uncontrolled urbanization. Yet very few studies have studied the interactions between urban environments and malaria. Additionally, no standardized urban land-use/land-cover has been defined for urban malaria studies. Here, we demonstrate the potential of local climate zones (LCZs) for modeling malaria prevalence rate (*Pf* PR_2−10_) and studying malaria prevalence in urban settings across nine sub-Saharan African cities. Using a random forest classification algorithm over a set of 365 malaria surveys we: (i) identify a suitable set of covariates derived from open-source earth observations; and (ii) depict the best buffer size at which to aggregate them for modeling *Pf* PR_2−10_.

Our results demonstrate that geographical models can learn from LCZ over a set of cities and be transferred over a city of choice that has few or no malaria surveys. In particular, we find that urban areas systematically have lower *Pf* PR_2−10_ (5%–30%) than rural areas (15%–40%). The *Pf* PR_2−10_ urban-to-rural gradient is dependent on the climatic environment in which the city is located. Further, LCZs show that more open urban environments located close to wetlands have higher *Pf* PR_2−10_. Informal settlements—represented by the LCZ 7 (lightweight lowrise)—have higher malaria prevalence than other densely built-up residential areas with a mean prevalence of 11.11%. Overall, we suggest the applicability of LCZs for more exploratory modeling in urban malaria studies.

## 1 Introduction

In sub-Saharan Africa, malaria transmission is maintained by mosquito vectors that are predominantly found in rural environment (Hay *et al* 2005, Machault *et al* 2010). But rapid and uncontrolled urbanization in sub-Saharan Africa (Union 2017, Wolff *et al* 2020) increased the amount of exposed urban inhabitants. The inherent appearance of informal and planned residential neighborhoods with their social inequalities (Eloundou-Enyegue and Giroux 2012, Obeng-Odoom 2015, Korah *et al* 2019), and the increasing areas allocated to urban agriculture and neighboring wetlands have led to spatial disparities in urban malaria risks (Klinkenberg *et al* 2005, Baragatti *et al* 2009, Dongus *et al* 2009, Kienberger and Hagenlocher 2014, Kabaria *et al* 2016). Understanding the interactions between the heterogeneous urban environments and malaria have thus become urgent and essential for tackling malaria burden in Africa (Georganos *et al* 2020).

Because of the complex nature of risk factors in urban environments most of urban malaria research has been constrained to the level of case studies and major review papers (e.g. Robert *et al* (2003), Hay *et al* (2005), De Silva and Marshall (2012)). Furthermore, few spatial modeling efforts of malaria— or its vectors—prevalence in urban environments have been done (e.g. Machault *et al* (2012), Borderon (2013), Kabaria *et al* (2016), Georganos *et al* (2020)). Additionally, malaria risk mapping initiatives at the global, continental or national level (Guerra *et al* (2006, Tatem *et al* 2008, Raso *et al* 2012, Noor *et al* 2014, Bhatt *et al* 2015) simplified urban settlements as a binary covariate, without considering their heterogeneities in forms and functions (Bennett *et al* 2013, Giardina *et al* 2015). As a consequence, there are to date no standardized approaches for classifying the urban environment for malaria studies. The development of such approaches is further hampered by scarce documentation on cities’ forms and functions in tropical Africa. To address this scarcity, novel and open source tools have been developed, offering an universal and simple representation of urban landscapes based on local climate zones (LCZs; Stewart and Oke (2012)). Currently, the World Urban Database and Access Portal Tool (WUDAPT; Bechtel *et al* (2015), Ching *et al* (2018)) is leading the way for acquiring a city- to continental-wide land-use/land-cover (LULC) classification based on LCZs, thereby offering a detailed representation of the urban heterogeneities (Bechtel *et al* 2019, Demuzere *et al* 2019a, 2019b). LCZs describe an urban LULC using 10 urban classes and 7 natural ones. Each class is explanatory of a peculiar urban typology and its inherent climate. They are therefore defined in terms of impervious and pervious coverage, building densities and heights, anthropogenic heat fluxes and heat storage capacities (Stewart and Oke 2012). While the latter two are of less direct importance for malaria studies, they affect the vector’s survival capacity via their influence on urban climates (Gething *et al* 2010, 2011, Dalrymple *et al* 2015). Consequently, Brousse *et al* (2019) proposed the use of LCZs to relate urban climates to urban malaria risk and added a natural LCZ for that purpose: LCZ wetlands (LCZ W). With LCZs gaining in popularity for urban design and health studies (Middel *et al* 2014, Geletic *et al* 2018, Aminipouri *et al* 2019, Vandamme *et al* 2019), we hypothesize that they could be used as an universal and standard LULC classification for urban malaria studies in tropical Africa.

In this study we: (i) define a set of predictive variables obtained from LCZs and freely-accessible satellite remote sensing data to study malaria prevalence across tropical African cities; (ii) identify the spatial scale that is most suitable for an exploratory modeling of the heterogeneous urban environments’ influences on malaria prevalence; (iii) evaluate whether the information obtained from the set of predictive variables, and more specifically from LCZs, is transferable across African cities to study malaria prevalence; and finally (iv) predict malaria prevalence in multiple tropical African cities to analyze its systematic spatial patterns. We analyze the results to show the added value of LCZs for urban malaria studies and discuss its potential use for future research.

## 2 Data and methodology

### 2.1 Malaria surveys: data type and filtering

**Figure 1:**
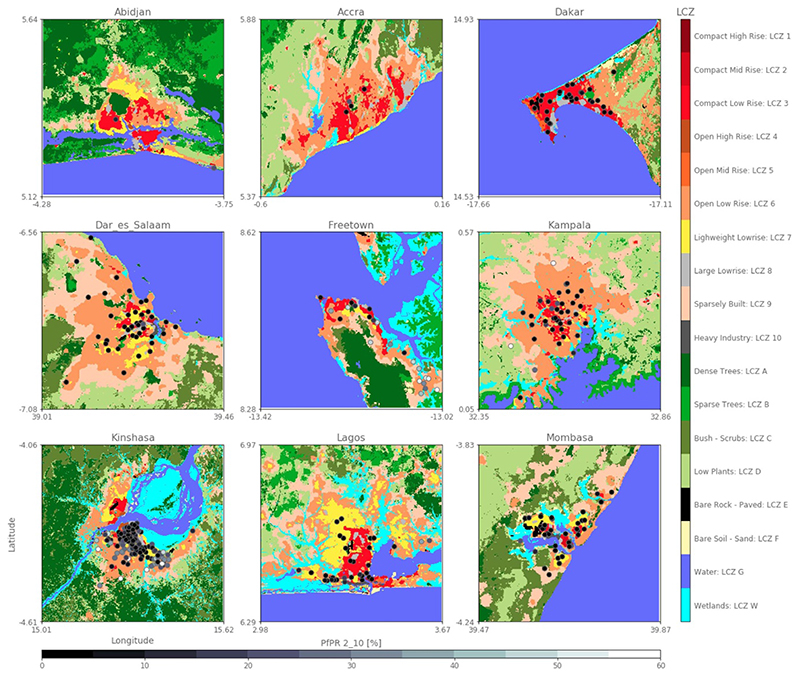
Accurately geolocalized malaria surveys and their respective *Pf*PR_2−10_ values in % plotted over the LCZ map of each selected city.

Data on malaria prevalence has been assembled over several years for multiple cities to provide a comprehensive overview of malaria infection risk across African cities (Snow *et al* (2017); http://doi:10.7910/DVN/Z29FR0). Malaria prevalence—or the *Plasmodium falciparum* parasite rate—is here defined as the fraction of examined individuals tested positive during a single cross-sectional survey for malaria. *Plasmodium falciparum* parasite rate is usually standardized for children aged 2–10 (hereafter referred to as *Pf* PR_2−10_; Smith *et al* (2007)) to enable comparison among surveys that have different age ranges’ targets. The Pull & Grab-based algorithm (Pull and Grab 1974) was considered the best by Smith *et al* (2007) for calculating *Pf* PR_2−10_. As our goal is to study the impact of urban environments on *Pf* PR_2−10_, the work solely focuses on accurately geolocated (with GPS coordinates or with the location validated in Google Earth; Georganos *et al* (2020)) survey estimates of *Pf* PR_2−10_ with coherent metadata recorded with at least 20 individuals sampled between 2005 and 2015 and who were aged below 18 years. In this way, we make sure that the standardization proposed by Smith *et al* (2007) includes enough examined people, while concentrating on children and adolescents with reduced mobility. This also avoids the inclusion of positive adults in the standardization, who tend to be confronted to a variety of urban environments because of their daily migrations (Andreasen *et al* 2017). This results in a sub-selection of 385 surveys covering nine cities (see figure 1 and table S1) and with a rounded average amount of 69 examined people. These surveys are composed of random selection of schools and communities across the urban environment. The final selection consists of (see figure S1): Abidjan (Ivory Coast), Accra (Ghana), Dakar (Senegal), Dar Es Salaam (Tanzania), Freetown (Sierra Leone), Kampala (Uganda), Kinshasa (Democratic Republic of Congo), Lagos (Nigeria) and Mombasa (Kenya). The rounded averaged amount of examined people per city is of 59, 104, 46, 79, 27, 71, 65, 94 and 85, respectively. All nine cities are: (i) endemic for malaria, (ii) metropolises of more than 1 M inhabitants, (iii) built at latitudes between 10.0°S and 20.0°N, and (iv) subject to the seasonal shifts of the inter-tropical convergence zone.

### 2.2 Mapping LCZs

We mapped the nine cities in the form of LCZs since they were not publicly available on the WUDAPT portal (Ching *et al* 2018). Our mapping method is based on Demuzere *et al* (2019a), Demuzere *et al* (2019b), applying Google’s Earth Engine (GEE, Gorelick *et al* (2017)) random forest (RF) classification algorithm (Breiman 2001) on a variety of earth observation datasets. The model is trained using a set of training areas (polygons) that are digitized over Google Earth images for each city. These training areas have been gathered during a mapathon held at the Université Libre de Bruxelles on the 15th of November 2019. To evaluate the model we bootstrap the RF model 25 times using 70% of the data for training and evaluating against the remaining 30%. We perform this evaluation in an iterative way where the original sets of training areas are reworked after each iteration until we obtain a satisfactory overall accuracy (OA) measure of at least 50%, as proposed by Bechtel *et al* (2019). Other measures are employed for assessing the mapping quality: the OA for the urban LCZ classes only (OA_*u*_), the OA of the built versus natural LCZ classes only (OA_*bu*_), and the weighted accuracy (OA_*w*_) (see Bechtel *et al* (2017), Bechtel *et al* (2020)). Optimal F1 accuracies per LCZ—‘*which represents the arithmetic mean of the class-wise F1 values, which are calculated as the weighted harmonic mean of the user’s (UA) and producer’s accuracy (PA)*’; in Verdonck *et al* (2019), pp. 6—are also desirable but can be highly influenced by their respective number of training areas.

The predictive variables used in Demuzere *et al* (2019a) are expanded with Sentinel 1 Gray Level Cooccurrence Matrix textures with a 11 by 11 window size to better capture the heterogeneities of built up surfaces (Forget *et al* 2018) as well as Sentinel 2 red edge bands to improve the mapping of LCZ wetlands (Forkuor *et al* 2018, Kaplan and Avdan 2018) (for a complete list of the variables used in the LCZ mapping see table S2)).

**Figure 2:**
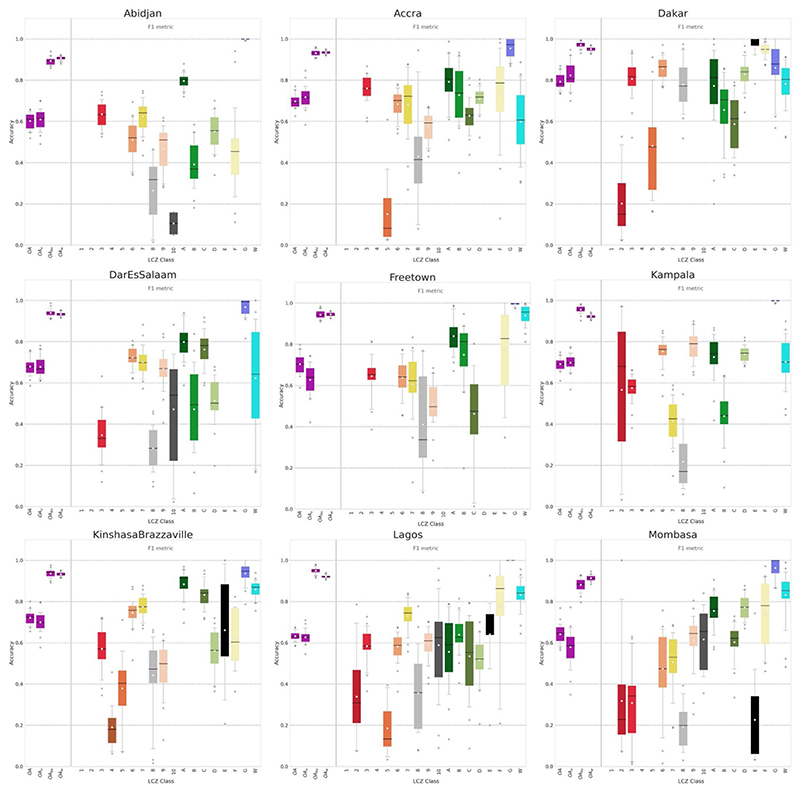
Boxplots of different accuracy indicators’ values for each city. In purple are, from left to right, the overall accuracy OA, the overall accuracy of urban LCZ OA_*u*_, the overall accuracy of the built up against the natural environment OA_*bu*_ and the weighted accuracy OA_*w*_. F1 accuracies per LCZ are given following the same LCZ color scale than in figure 1. Zoomed in boxplots per city are provided in supplements (figures S2(a)–(h)).

Once all OAs are above the recommended value of 0.5 (figure 2), all training areas are used to map each city in the form of LCZ at 100 m resolution. Reaching this value for all nine cities took about 10 working days at full time by an expert (see Brousse *et al* (2020a) for more information on the challenges for mapping LCZ in sub-Saharan Africa). As single pixels do not constitute an LCZ class, and granularity is often present in the raw LCZ maps, the raw LCZ maps are post-processed using a Gaussian filter (Demuzere *et al* 2020). Compared to the default majority post-classification filter with a radius of 300 m, this Gaussian approach takes into account typical patch sizes for each LCZ class (e.g. rivers are often more narrow than residential neighborhoods). This way, informal settlements, river channels, and wetlands, for example, are retained after filtering (figure 1).

### 2.3 Acquiring remotely sensed predictive variables

As previous studies demonstrated, rainfall, near-surface and surface temperatures, LULC, surface moisture, distance to breeding sites, vegetation indices and elevation variables are commonly used for mapping malaria prevalence (see Weiss *et al* (2015), Parselia *et al* (2019)). Here, we define open accessibility to the data, exhaustive coverage, and horizontal and temporal resolutions as major criteria for choosing our data sources. This means that we derive our covariates from freely-available remotely sensed earth observation products without using in-situ information. We decide to exclude both near-surface and surface temperature from the covariates as (i) spatially explicit urban near-surface temperatures are difficult to obtain from remotely sensed data only (Zhou *et al* 2019, Venter *et al* 2020) and (ii) urban land surface temperatures cannot suffice as they are known to be subject to high uncertainties— the latter being mostly related to the complex three-dimensional landscape of cities (Voogt and Oke 1998, Voogt and Oke 2003). Moreover, our cities are all located in a tropical climate—defined by a monthly mean temperature that does not decrease below 18 °C—that makes their climate environments all suitable for transmission of malaria across the year (also see figure 3(b) from Gething *et al* (2011)).

Hence, we gather: (i) LCZ maps at a native resolution of 100 m for each city representative of years 2017–2019—assuming that the urbanization rate over the past 14 years was not sufficient to impact malaria prevalence in the studied cities—to capture the generic influence of common LULC features across cities on *Pf* PR_2−10_ (e.g. distance to LCZ wetlands introduced in Brousse *et al* (2019) or proportions of densely built LCZs); (ii) averaged normalized difference vegetation and wetness indices (NDVI and NDWI, respectively) and their temporal standard deviations (*σ*) over the period 2005–2019 from the Landsat 5 and 8 libraries (at a native resolution of 30 m) to capture the local effect of vegetation and soil moisture on *Pf* PR_2−10_; (iii) averaged elevation of 30 m pixels in 100 m pixels for the year 2000 from the Shuttle Radar Topography Mission digital to capture the differences of *Pf* PR_2−10_ across cities based on their elevation and also within cities (e.g. low-lying and elevated areas); and (iv) yearly averaged maximum, minimum and mean monthly precipitation at 0.1° resolution over the period 2005–2017 from the multi-source weighted-ensemble precipitation data-set (MSWEPv2; Beck *et al* (2017), Beck *et al* (2019)) to capture the influence of the seasonal amplitude of precipitation on *Pf* PR_2−10_ across cities. All data, apart from the MSWEP product, are pre-processed on GEE and extracted at the LCZ resolution of 100 m.

### 2.4 Selection of predictive variables and buffer sizes

**Figure 3:**
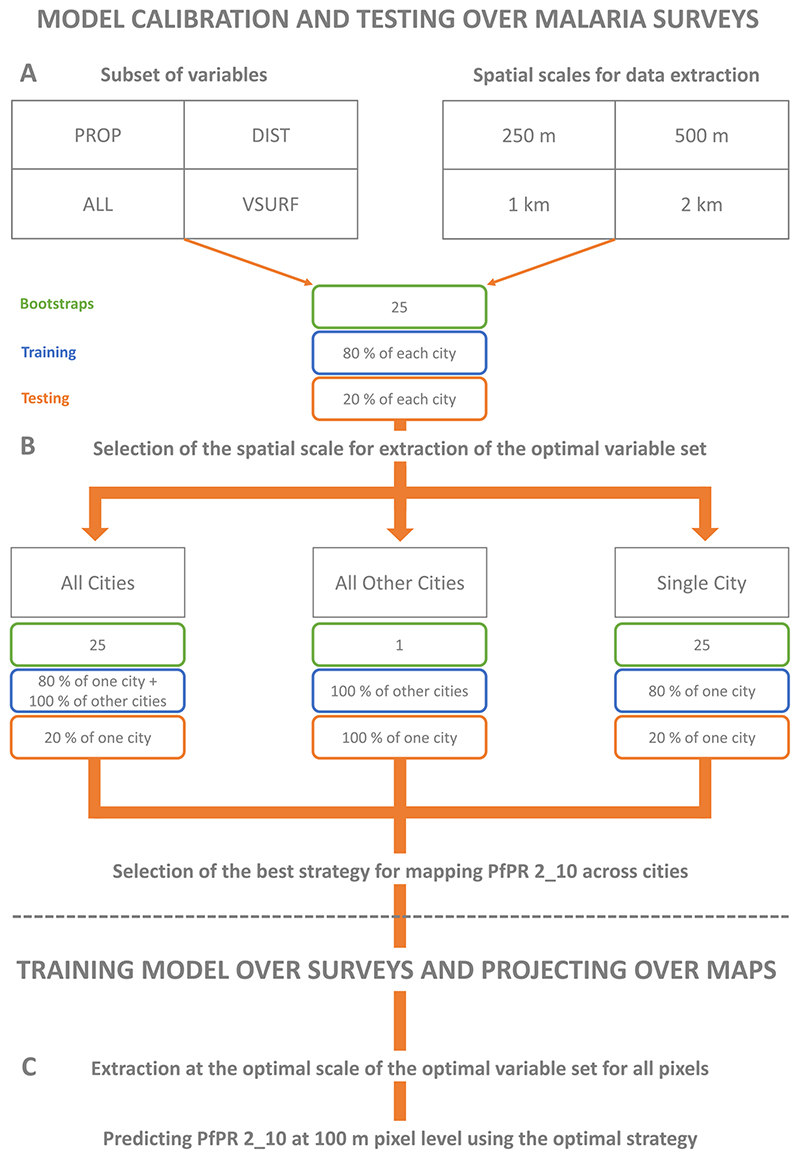
Schematic representation of the workflow followed in this study for modeling *Pf* PR_2−10_ across nine African cities.

Four buffer radii centered over the surveys’ locations are tested for predicting malaria prevalence using the above-mentioned variables (figure 3(A)): 250 m, 500 m, 1 km and 2 km. This step permits the definition of an optimal scale at which relations between the heterogeneity of urban environments and *Pf* PR_2−10_ in cities can be studied. This step is necessary as both the examined people and the vector can move throughout the urban environment. Yet, as our sample is filtered to keep only schools and community surveys focusing on children with lowered mobility, and since mosquitoes tend to migrate only over a few hundreds of meter to few kilometers in urban areas for feasting (Byrne 2007, Machault *et al* 2010, Verdonschot and Besse-Lototskaya 2014), we do not define a buffer larger than 2 km. We chose to use an RF model because it (i) efficiently handles noisy and/or multisource data, (ii) focuses on average relationships between the covariates and the predicted variable and (iii) manages data that are coming from temporally and spatially heterogeneous surveys (Georganos *et al* 2019).

For the normalized difference indices and the elevation variables we extract the mean of the buffer. For the precipitation data, we assign the underlying value to the centroid of the buffer because the horizontal resolution of 0.1° is greater than the maximum buffer radius of 2 km. For the LCZ information, we derive the proportions of LCZ contained within the buffer, and the averaged minimum distance of points within the buffer to other LCZ classes outside of the buffer. Because of the similarities between some LCZ—as demonstrated by Bechtel *et al* (2017), Bechtel *et al* (2020)—in terms of densities and land cover types, we chose to merge some of them. Additionally, the amount of surveys comprised in high- and mid-rises LCZ classes was small (see table S1, which is available online at https://stacks.iop.org/ERL/15/124051/mmedia), supporting the merging of similar classes to ease the interpretation. The same rationale was applied for natural classes. LCZ were thus merged as follows: LCZ compact (compact high-, mid- and low-rise: LCZ 1, 2 and 3), LCZ open (open high-, mid- and low-rise: LCZ 4, 5 and 6), LCZ industrial (large lowrise and heavy industry: LCZ 8 and 10)), LCZ trees (dense trees and open trees: LCZ A and B)), LCZ lowland (bush—scrubs and lowland: LCZ C and D)). Remaining LCZ classes (LCZ 7: lightweight lowrise—also considered as informal settlements, LCZ 9: sparsely built; LCZ G: water—same as in the LCZ classification but constrained to open and running waters, and LCZ W: wetlands—introduced in Brousse *et al* (2019) as an important variable for malaria epidemiological studies) are retained as standalone variables for the *Pf* PR_2−10_ model. LCZs E and F (bare rock or paved, and bare soil or sand, respectively) are excluded as predictive variables because they are constrained to beaches and airports and are thus not representative of major features in the urban environment.

Additionally, the sensitivity of the *Pf* PR_2−10_ model is tested with respect to its input features. We used four different sets of input features (figure 3(A)): (i) all predictive variables (ALL), (ii) all the variables excluding the distances to LCZs (PROP), (iii) all the variables excluding the LCZ proportions (DIST) and (iv) the most important variables for each buffer size given by the interpretation step of the VSURF package in R (VSURF; Genuer *et al* (2015)).

All the surveys from each city are merged together to test the most predictive set of variables, for all cities and per buffer size. We then run the RF regression model (Breiman 2001) 25 times by following a bootstrapping procedure that randomly selects 80% for training the model and 20% of the data for testing. In addition, the random selection is stratified according to cities’ amount of surveys ensuring that all cities are always used for training and testing the model in a coherent manner across each bootstrap. Based on root-mean squared error (RMSE), mean absolute error (MAE) and the coefficient of determination (*R* ^2^)—which are calculated on the 20% remaining for testing—an optimal set of variables at a determined buffer size is used for training the RF model and modeling *Pf* PR_2−10_ for each city.

### 2.5 Are RF models using LCZ transferable across different cities for modeling *Pf* PR_2−10_ ?

Once the optimal set is defined, we test if models that are built on multiple cities using LCZs can be transferred over single cities under consideration to model and study their *Pf* PR_2−10_.

We first compare the model performances from the best set of variables with and without a dummy variable that refers to each city—numbers from 1 to 9 in our case. If model performances are significantly better by integrating these dummies, local features that are not considered in this study—for example socio-economical or temperature parameters— would play a more important role than how and where cities are built for modeling *Pf* PR_2−10_. Transferring the urban environmental information from one city to another might thus not be possible. Second, we evaluate how the RF model is capable of accurately transferring cities’ information for modeling *Pf* PR_2−10_ in a single city (figure 3(B)) by comparing RMSE, MAE and *R* ^2^ from three different modeling strategies where we: (i) use all the other cities’ data and test over the held-out city. This strategy is called ‘All Other Cities’; (ii) bootstrap 25 times using only the data available for the specific city under consideration with a random selection at each bootstrap of 20% of the data for testing and 80% for training. This strategy is called ‘Single City’; and (iii) test the added value of complementary information from other cities for more accurate predictions in a single city. For this, we bootstrap 25 times using all the data from the other cities, in addition to a random selection at each bootstrap step of 80% of the data from the city to be mapped for training. The remaining 20% of the data from the city to be mapped is kept at each step for testing. This strategy is called ‘All Cities’.

### 2.6 Mapping *Pf* PR_2−10_ per LCZ

After defining the most optimal training set and buffer size for modeling *Pf* PR_2−10_ across all cities, we map *Pf* PR_2−10_ at a horizontal resolution of 100 m for each city. Afterwards, we compare the outcomes between cities (e.g. cities that have a higher prevalence than others) and subsequently quantify the *Pf* PR_2−10_ per LCZ class across all cities to show which LULC classes could systematically at higher risks of prevalence in tropical Africa (figure 3(C)).

## 3 Results

**Figure 4:**
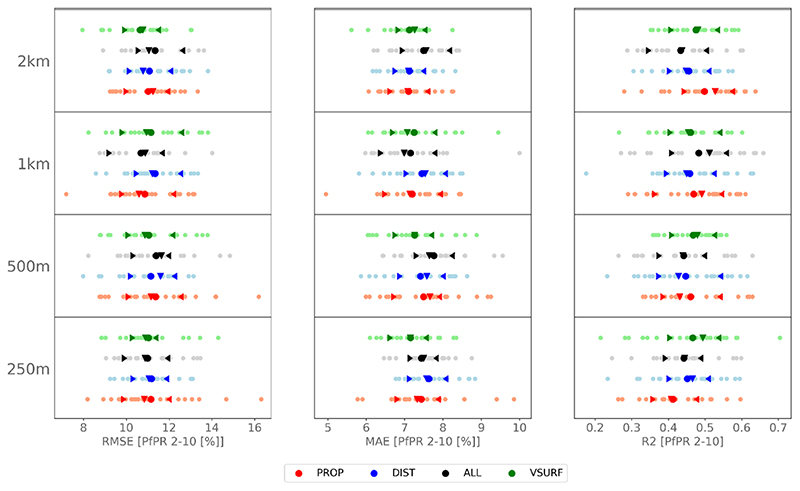
RMSE, MAE and *R* ^2^ scores for the 25 bootstraps of each buffer size (250 m, 500 m, 1 km and 2 km) using the four different sets of variables (ALL, PROP, DIST and VSURF). Averaged scores are in thick points, median are represented by the down triangle, and the inter-quartile range is given by the summits of the left and right carets.

The mean *Pf* PR_2−10_ over the whole data set is of 10.45% with a *σ* of 14.96%. We find that our models depict averaged statistical scores ranging from 10.64 [% PfPR_2−10_] to 11.39 [% PfPR_2−10_], 7.10 [% PfPR_2−10_] to 7.76 [% PfPR_2−10_], and 0.41 to 0.5 for RMSE, MAE, and *R* ^2^, respectively (figure 4). With maximum differences of 0.75 [% PfPR_2−10_] for RMSE, 0.66 [% PfPR_2−10_] for MAE, and 0.09 for *R* ^2^, the sensitivity to buffer sizes and predictors appears to be rather low. The distribution of the predictions seems to follow a quasi-normal distribution, with median RMSE, MAE and *R* ^2^ always close to the mean. Also, differences between *σ* are not significant, according to a Wilcoxon rank-sum test.

We therefore opt for a buffer size of 1 km for an exploratory modeling of *Pf* PR_2−10_ across all cities using all predictive variables (ALL; figure 4). This buffer size and variables set gives the 2nd, the 5th and the 2nd best mean RMSE, MAE and *R* ^2^ respectively, while still offering a full set of variables that can explain *Pf* PR_2−10_. According to the variable importance, we find that the ten most important variables are precipitation, normalized difference indices and their standard deviation, elevation and distances to LCZ compact, LCZ informal, and LCZ industrial. All the other variables derived from LCZ, apart from the proportion of LCZ industrial, are of relative importance and contribute to an increase in model’s performance (figure S3).

**Figure 5:**
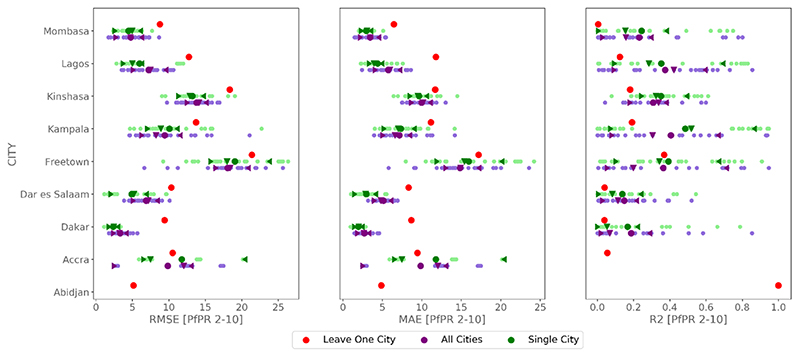
RMSE, MAE and *R* ^2^ scores for each city using *All Other Cities* strategy (in red), *All Cities* strategy (in purple) and *Single City* strategy (in green) using buffers of 1 km. Scores for each bootstrap are in light colors while thick points are the averaged scores, median are represented by the down triangle, and the inter-quartile range is given by the summits of the left and right carets.

The inclusion of dummies referring to each city leads to a slight deterioration of model performance when using all variables (ALL) obtained within a 1 km buffer. In particular, this leads to a reduction of mean *R* ^2^ by 3.84% and an increase of mean MAE and mean RMSE by 5.04% and 5.53%, respectively. Extending the single city data with information from other cities (*All Cities*) results in similar performances compared to using single city data only (*Single City*). In addition, the *All Cities* tends to reduce the uncertainty between each bootstrapping step (figure 5). In comparison to the two other strategies, using the *All Other Cities* strategy results in an absolute deterioration of the model performance by 4.18 [% *Pf*PR_2−10_] for RMSE, 3.16 [% *Pf* PR_2−10_] for MAE and 0.16 for *R* ^2^, in average. But, when comparing model performances per city (e.g. Freetown’s statistical indicators against Kampala’s) relative orders are respected. These results overall confirm that the information obtained by the model over other cities can be transferred for modeling *Pf* PR_2−10_ in the city under consideration.

**Figure 6:**
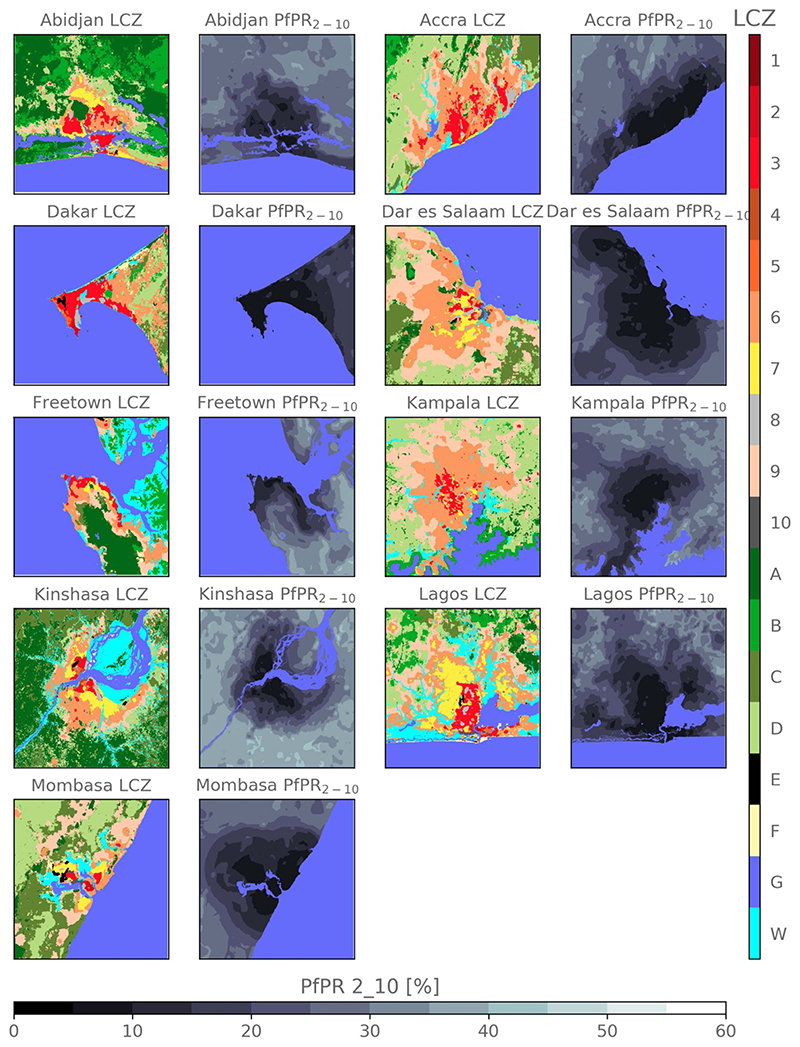
Modeled *Pf* PR_2−10_ in percentage (%) per city using single city data and all other cities’ data in addition (*All Cities*, right panel). The left panel shows the LCZ map for visually relating *Pf* PR_2−10_ maps to the LULC of each city. Open water and rivers (LCZ (G) are masked out in the *Pf* PR_2−10_ maps.

Considering all the above-mentioned results, we are able to map *Pf* PR_2−10_ in each city at a horizontal resolution of 100 m using all predictive variables (ALL) gathered in a 1 km buffer size around each pixel. We train the RF model over all 365 surveys (*All Cities*). Results highlight that urban areas have *Pf* PR_2−10_ values between 5% and 30%, while this is between approximately 15% to 40% for rural areas (figure 6). The gradient from the urban center to the rural areas is different between each city suggesting that the endemicity of each local environment is well captured by the model. The bigger differences between urban and rural areas are located in Kinshasa, while cities like Dakar and Mombasa have small urban to rural gradients of *Pf* PR_2−10_.

**Figure 7:**
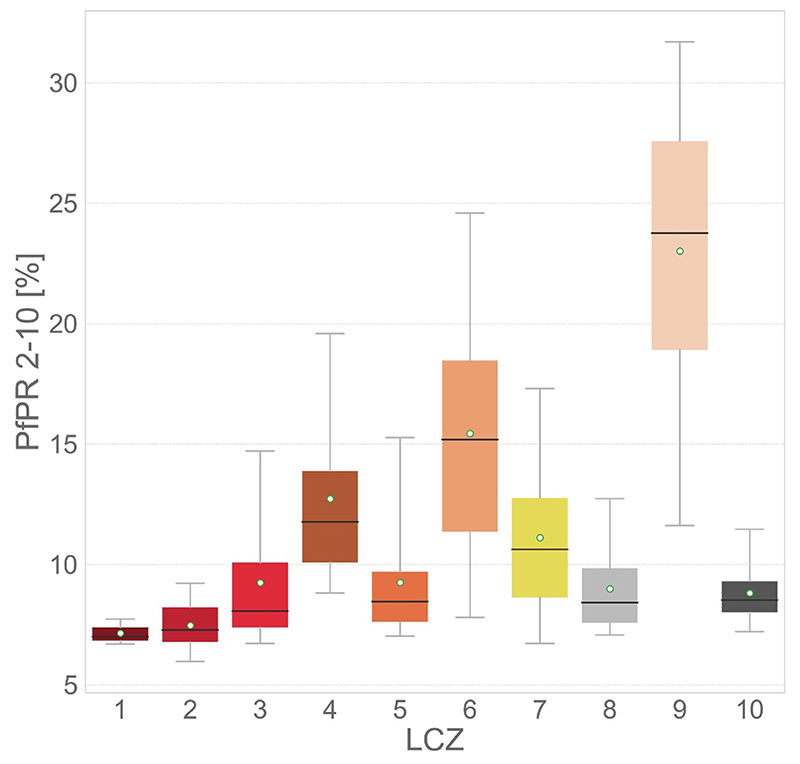
Modeled *Pf* PR_2−10_ in percentage (%) per urban LCZ across all cities using single city data and all other cities’ data in addition (*All Cities*). The distribution is represented in the form of boxplots where boxes are the interquartile range, whiskers the 5 to 95th percentile, black horizontal lines the median, and white points the mean.

When separating back the merged LCZ classes and looking at the modeled *Pf* PR_2−10_ per LCZ (figure 7), we can see that dense LCZs (LCZ 1–3) have the lowest *Pf* PR_2−10_ values with mean *Pf* PR_2−10_ values of 7.14%, 7.47% and 9.24%, respectively. Higher mean *Pf* PR_2−10_ are observed in open low-rise (LCZ 6) and sparsely built (LCZ 9) environments with values of 15.44% and 23.0%, respectively. In addition, very densely built informal settlements (LCZ 7—lightweight lowrise) have a higher mean *Pf* PR_2−10_ than other densely built classes with a value of 11.11%. Industrial areas (LCZ 8—large lowrise, and LCZ 10—heavy industry) have *Pf* PR_2−10_ values below 10%.

## 4 Discussion and conclusions

In this study, we demonstrate that the universal LCZs LULC classification can be used for modeling and studying malaria prevalence (*Pf* PR_2−10_) across tropical African cities. In particular, we show that LCZ can efficiently help to understand the influence of urban environments on *Pf* PR_2−10_ and that this information can be transferred to other cities to study urban *Pf* PR_2−10_ in distinct urban areas in tropical Africa. Our results therefore suggest that geographical models could be trained on other cities to model *Pf* PR_2−10_ in a selected city that has no malaria survey—yet acknowledging a probable deterioration of the model performance. Because LCZs are designed to represent urban forms and functions across the world in a generic way (Stewart and Oke 2012), they allow for a standardization of the urban LULC information that enables modeling of *Pf* PR_2−10_’s spatial heterogeneities in urban and peri-urban environments. Indeed, our modeling performances are in line with previous spatial modeling of *Pf* PR_2−10_ that modeled the spatial distribution of *Pf* PR_2−10_ in the cities of Dar Es Salaam and Kampala (Kabaria *et al* 2016, Georganos *et al* 2020). In these studies, RMSEs are ranging between 6.02 [% *Pf* PR_2−10_] and 16.02 [% *Pf* PR_2−10_] for the city of Dar Es Salaam, depending on the covariates that were used, while the only mapping over Kampala—that used very-high resolution satellite imagery—had a median RMSE of 5.45 [% *Pf* PR_2−10_]. In our study, the mean RMSE is 6.86 [% *Pf* PR_2−10_] and 9.43 [% *Pf* PR_2−10_], respectively, for the two latter cities. This shows that a RF regression model can be trained to predict *Pf* PR_2−10_ at a horizontal resolution of 100 m by including the variability of the urban environment in buffers of 1 km radius around each malaria survey. These model outputs at high resolution should however be constrained to exploratory purposes and not be considered as finite maps of *Pf* PR_2−10_.

**Figure 8:**
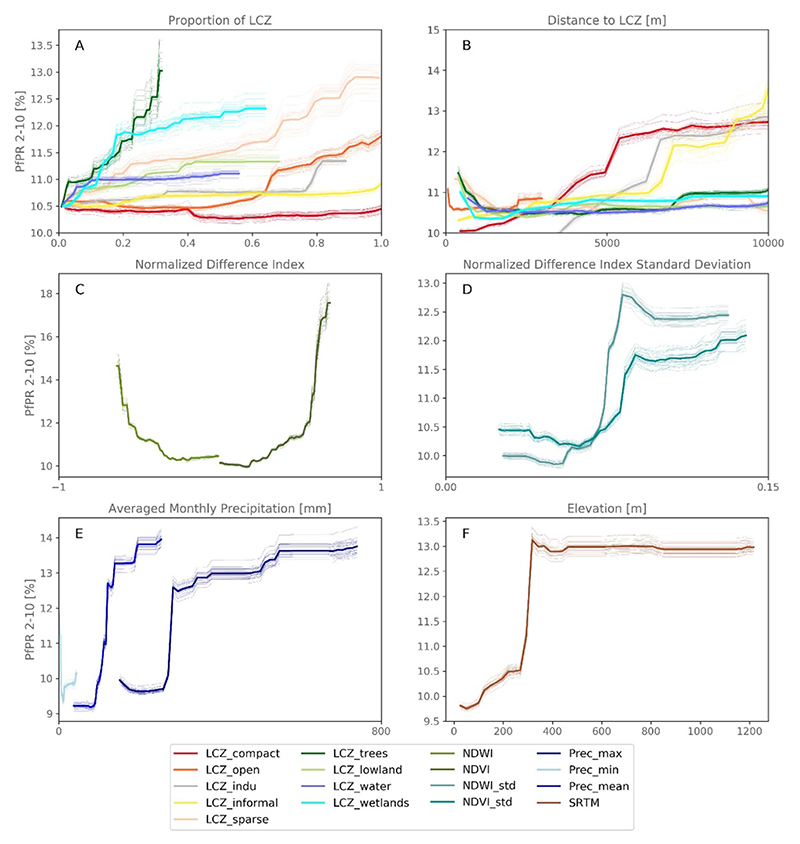
Averaged partial dependence plots (thick lines) over 25 model runs (light lines) for all variables used in the study. Variables are grouped by type: proportions of Local Climate Zones (LCZs; (A), distances to LCZs (B), normalized difference indices (NDI; (C), standard deviation of NDI (D), precipitation (E) and elevation (F).

To illustrate the latter, partial dependence plots (figure 8)—that characterize the response of *Pf* PR_2−10_ to a given explanatory variable—show that an increase in proportion of open LCZ (e.g. LCZ open or LCZ sparse) is positively correlated to an increase in *Pf* PR_2−10_ while more dense urban areas (LCZ compact) leads to lower *Pf* PR_2−10_. In addition, a slight increase of wetlands coverage from 0% to approximately 20% in the buffer zone leads to an increase in *Pf* PR_2−10_ from 10.5% to 12% (figure 8(A)). Finally, when looking at the partial dependence plots of normalized difference indices, precipitation and elevation, we can see that cities that are embedded in greener and wetter environments, far from the oceans, tend to have higher malaria prevalence (figures 8(C)–(F)). This is however only true for peri-urban and rural environments as our maps highlight similar *Pf* PR_2−10_ in densely built urban environments. The latter could explain why distances to densely built urban neighborhoods and greenness indicators like NDVI are covariates of high importance.

It is indeed commonly accepted that dense urban areas have lower malaria prevalence than surrounding rural environments and that peri-urban areas are also at higher risk (Robert *et al* 2003, Hay *et al* 2005, Kabaria *et al* 2017). Previous case studies also concluded that informal settlements have a higher prevalence than planned residential neighborhoods (De Castro *et al* 2004, Mukasa *et al* 2014, Georganos *et al* 2020). One potential explanation could be that informal settlements are forced to be built around unsanitary places, like wetlands, which can be used for urban agriculture (Kabumbuli and Kiwazi 2009, Vermeiren *et al* 2013). However, wetlands and urban agricultural fields are known to increase vectorial capacities (Afrane *et al* 2004, Dale and Knight 2008, Verdonschot and Besse-Lototskaya 2014). This is also depicted in our study, with urban settlements that are built close to wetlands—and this independent of their neighborhood typology—having higher *Pf* PR_2−10_. The results sustain the introduction of LCZ wetlands (LCZ W) proposed by Brousse *et al* (2019) for vector-borne disease studies.

Albeit the similarity of our conclusions to the already existing body of literature, none of these studies introduced a standardized LULC classification to study the relations between urban form and functions and malaria prevalence across tropical Africa. Our study suggests that LCZs a suitable tool for such purposes. Certainly, information on the urban environments alone does not suffice to explore the factors that explain the heterogeneous dispersal of malaria in cities. Part of the error depicted above may be related to the fact that although LCZs are similar in their building typologies across cities, they can still withhold disparate socio-economic dimensions that influence individuals’ vulnerabilities, for example. Moreover, our study does not integrate temperature variations as a limiting factor for malaria prevalence and should therefore only be considered representative of places where malaria is endemic throughout the year. For instance, additional information on urban meteorological variables at high resolution (e.g. Brousse *et al* (2020b), Van de Walle *et al* (2020)) could allow for a deepened understanding of the influence of urban heat, dry and wind islands on the vectorial capacity. Improved model performances and greater insights on the drivers of malaria risk in urban environments could also be obtained from additional data on health infrastructure, diurnal migrations and other socio-economic factors that are not included in this study (see Boyce *et al* (2019)). In addition, there are inherent limitations to the malaria data that we use in our study because our product is temporally aggregated to analyze spatial patterns of malaria. This means that national interventions that happened during our 11-year period (2005–2015) are not taken into account, and nor are infections imported from recent rural-to-urban migrations. Finally, our LCZ LULC maps are only representative of recent years (2017–2019), hence hampering the quantification of the effect of recent urbanization on malaria prevalence in tropical African cities.

Yet, it appears that at least part of the spatial distribution of *Pf* PR_2−10_ in African cities is related to how they are built. Such conclusion could not have been depicted without the details provided by the LCZ LULC classification. For instance, other products, like the MODIS Land Cover Type Product (MCD12Q1; Sulla-Menashe and Friedl (2018)) or the Global Human Settlement Layer derived from Landsat satellites (Pesaresi *et al* 2013), only offer a single urban class without information on the variety of the urban environments. Typically, informal settlements, that constitute a neighborhood typology with its inherent socio-economical dimensions, are captured by the LCZ mapping and are linked to higher *Pf* PR_2−10_. Nevertheless, higher *Pf* PR_2−10_ are found in more open urban environments (open low-rise; LCZ 6) and in rural environments (sparsely built; LCZ 9). Using LCZ as a standard LULC classification thus eases the comparison of common features in urban *Pf* PR_2−10_ between cities and could help decision makers to learn from other strategies for lowering *Pf* PR_2−10_ performed in other cities. Noteworthy, our study does not integrate population densities per urban classes because of their complex obtainment at high resolutions (Georganos *et al* 2020). This may further increase the disparities in malaria transmission risks between different urban environments. For instance, number of people infected in densely populated informal settlements may be higher than in sub-urban areas. In the end, we suggest that LCZs should further be studied for potentially helping mapping intervention strategies in Africa. Future work could also try to define a standardized urban LULC classification specific to the study of urban malaria prevalence; Local Malaria Zones, for example.

## Supplementary Material

Supplementary material for this article is available online

Supplementary Data

## Data Availability

The data that support the findings of this study are available upon reasonable request from the authors.
