# Supplementary material for: Can we use local climate zones for predicting malaria prevalence across sub-Saharan African cities?
Source: Environ Res Lett. Author manuscript; Available in PMC 2022 Feb 23. (PMC7612418; doi:10.1088/1748-9326/abc996)
Supplement: Supplementary Data [file EMS143224-supplement-Supplementary_Data.pdf]

Supplements

	LCZ 1<br>Compact High-Rise	LCZ 2<br>Compact Mid-Rise	LCZ 3<br>Compact Low-Rise	LCZ 4<br>Open High-Rise	LCZ 5<br>Open Mid-Rise	LCZ 6<br>Open Low-Rise
Abidjan (2)	0	0	2	0	0	0
Accra (4)	0	0	2	0	0	2
Dakar (33)	0	2	25	0	0	4
Dar es Salaam (46)	0	0	6	0	0	16
Freetown (23)	0	0	5	0	0	10
Kampala (40)	0	2	12	0	0	20
Kinshasa (148)	0	2	26	0	3	31
Lagos (34)	0	2	12	0	0	6
Mombasa (55)	0	2	14	0	0	13
**Total (385)**	0	10	104	0	3	102
	LCZ 7<br>Lightweight Lowrise	LCZ 8<br>Large Lowrise	LCZ 9<br>Sparsely Built	LCZ 10<br>Heavy Industry	LCZ A<br>Dense Trees	LCZ B<br>Sparse Trees
Abidjan (2)	0	0	0	0	0	0
Accra (4)	0	0	0	0	0	0
Dakar (33)	0	2	0	0	0	0
Dar es Salaam (46)	19	1	2	1	0	1
Freetown (23)	3	1	3	0	0	0
Kampala (40)	2	0	3	0	0	0
Kinshasa (148)	74	8	0	0	0	0
Lagos (34)	12	1	0	0	0	0
Mombasa (55)	13	0	3	2	1	0
**Total (385)**	123	13	11	3	1	1
	LCZ C<br>Bush / Scrubs	LCZ D<br>Low Plants	LCZ E<br>Bare Rock / Asphalt	LCZ F<br>Bare Soil	LCZ G<br>Water	LCZ W<br>Wetlands
Abidjan (2)	0	0	0	0	0	0
Accra (4)	0	0	0	0	0	0
Dakar (33)	0	0	0	0	0	0
Dar es Salaam (46)	0	0	0	0	0	0
Freetown (23)	0	0	0	0	0	1
Kampala (40)	0	0	0	0	0	1
Kinshasa (148)	0	1	0	0	0	3
Lagos (34)	0	0	0	0	0	1
Mombasa (55)	0	4	3	0	0	0
**Total (385)**	0	5	3	0	0	6

Table S1: Number of surveys per LCZ in each city and in the whole data set (Total). The number in parenthesis next to the city name represents the total amount of surveys available in that city.

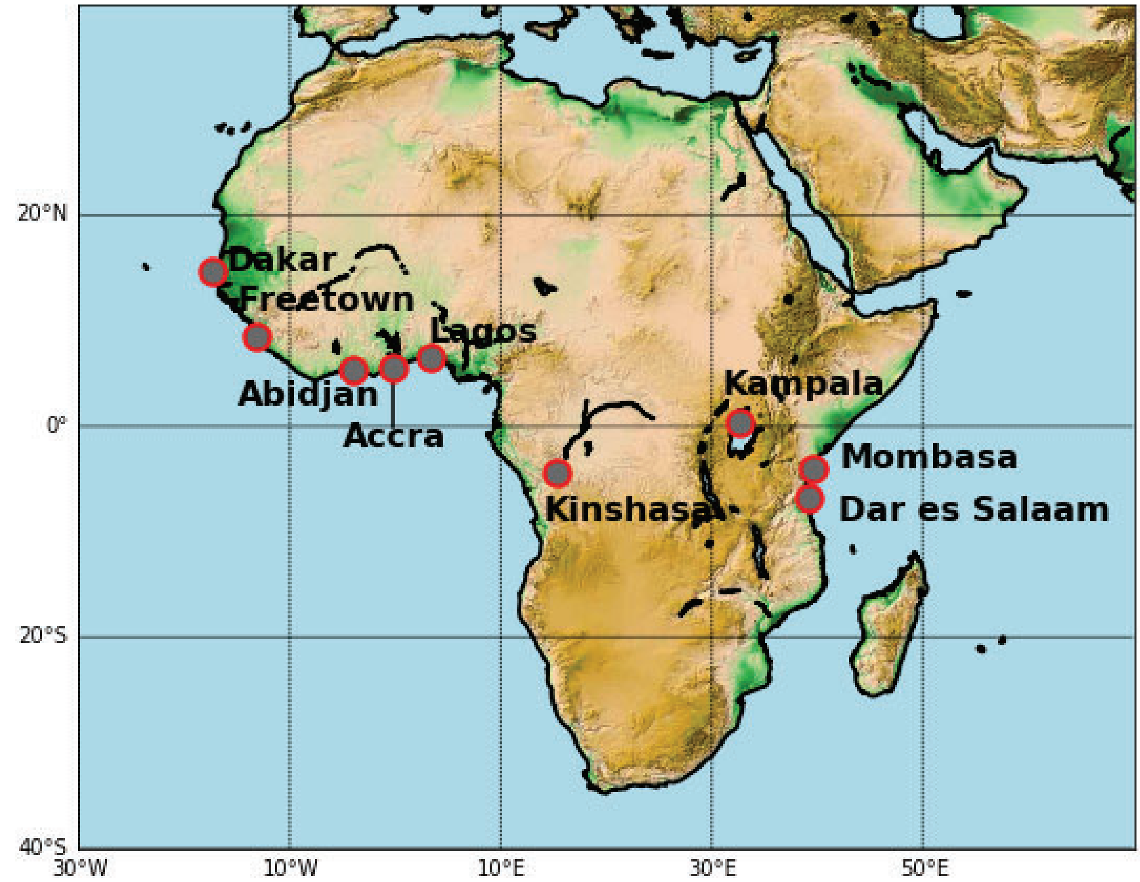

Figure S1: Location of the nine cities studies in this paper: Abidjan (Ivory Coast), Accra (Ghana), Dakar (Senegal), Dar Es Salaam (Tanzania), Freetown (Sierra Leone), Kampala (Uganda), Kinshasa (Democratic Republic of Congo), Lagos (Nigeria) and Mombasa (Kenya)

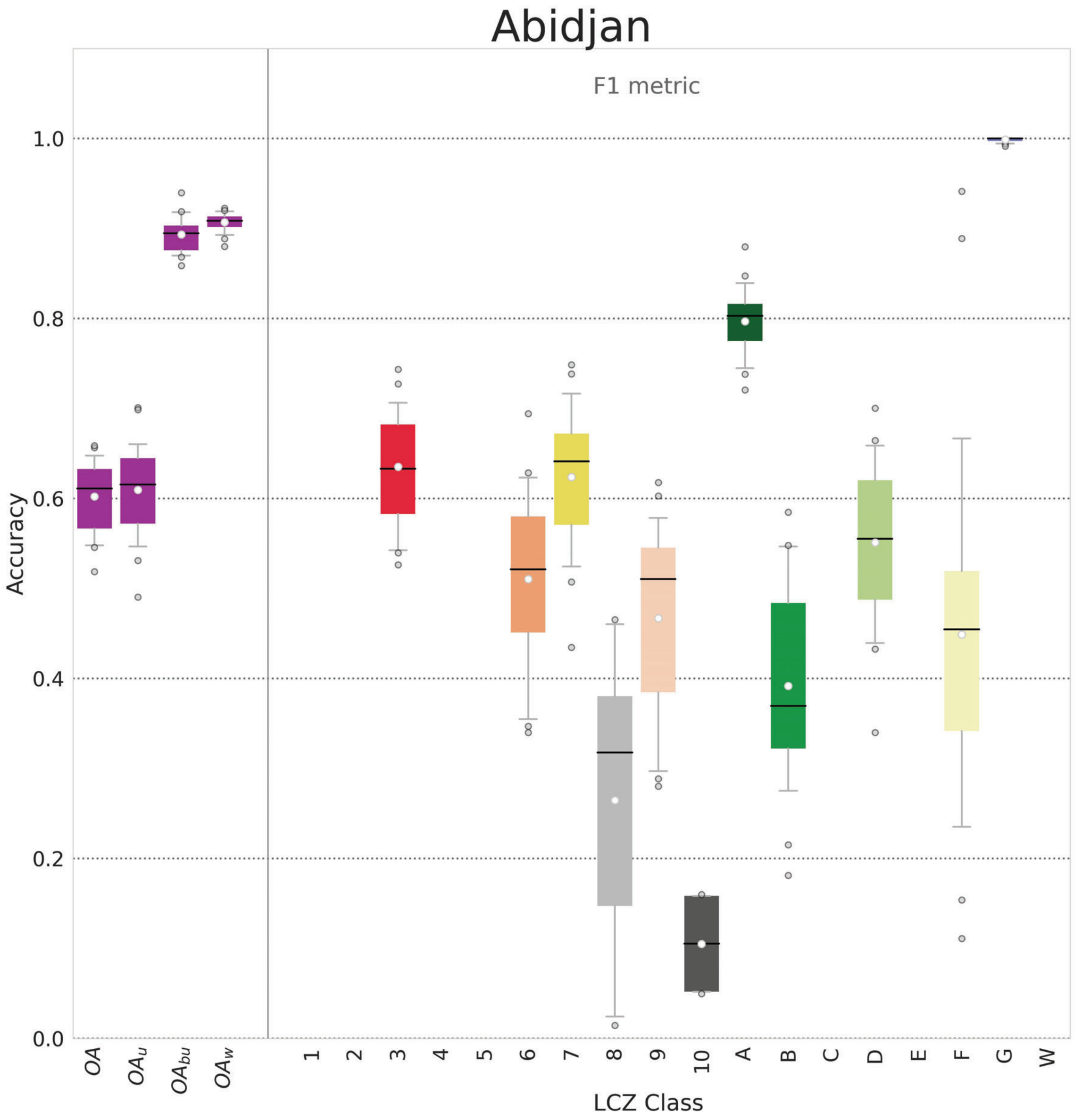

Figure S2a: Boxplot of different accuracy indicators' values for Abidjan. Details about accuracy indicators are given in Section 2.2. Subsequent Fig. S2b to Fig. S2h are identical to this one but for the other eight cities.

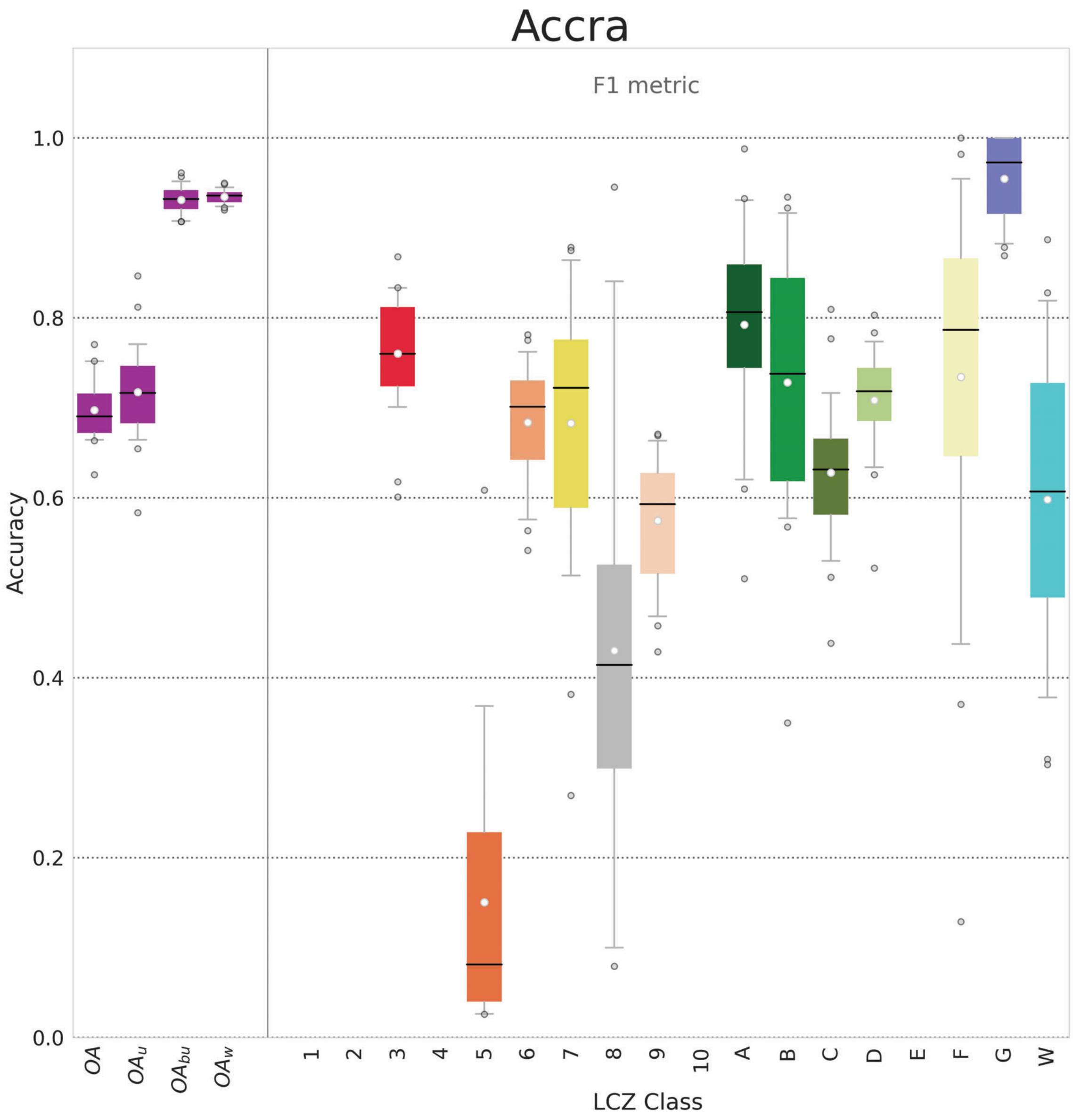

Figure S2b: Same figure as Fig. S2a but for Accra

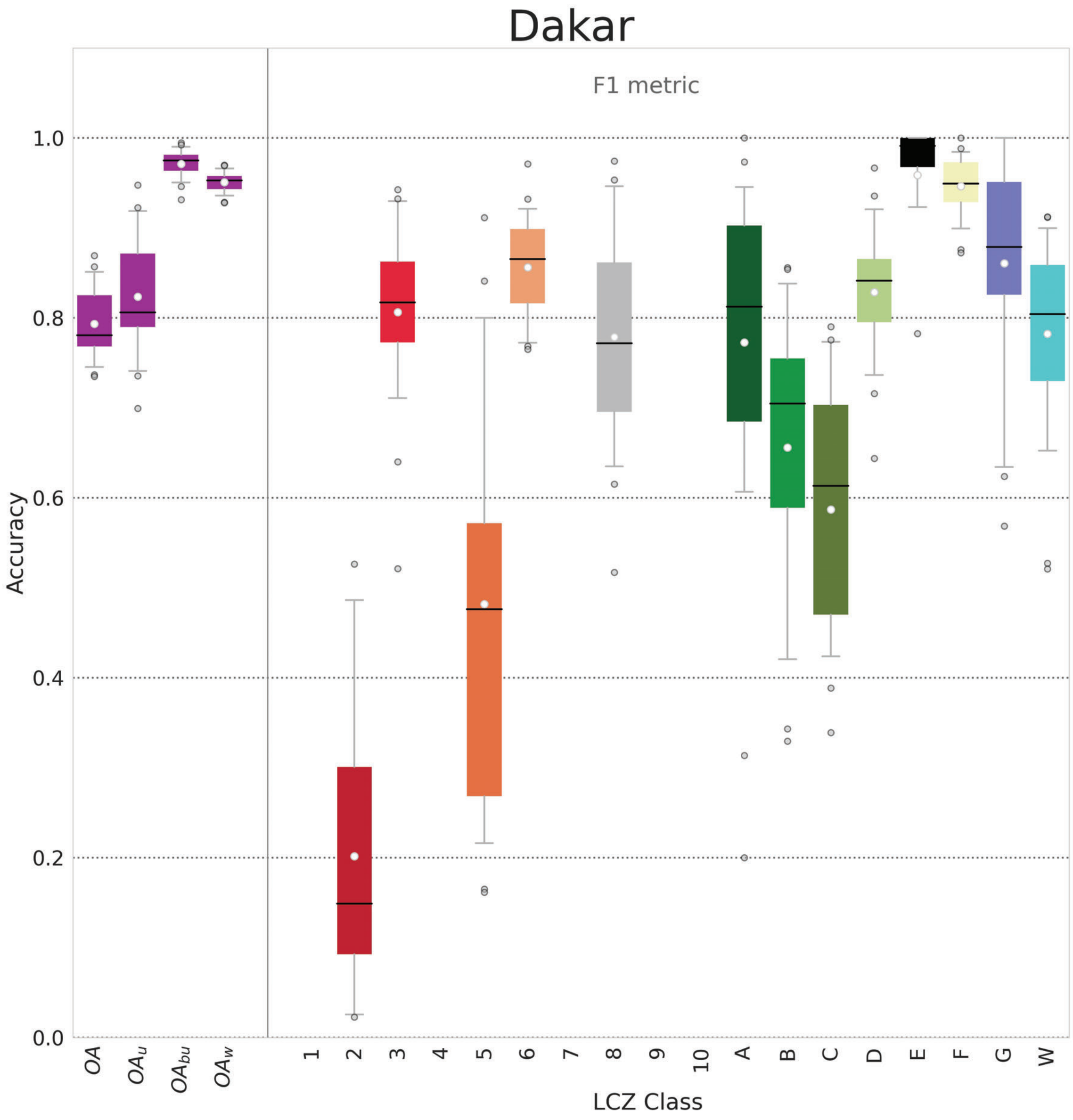

Figure S2c: Same figure as Fig. S2a but for Dakar

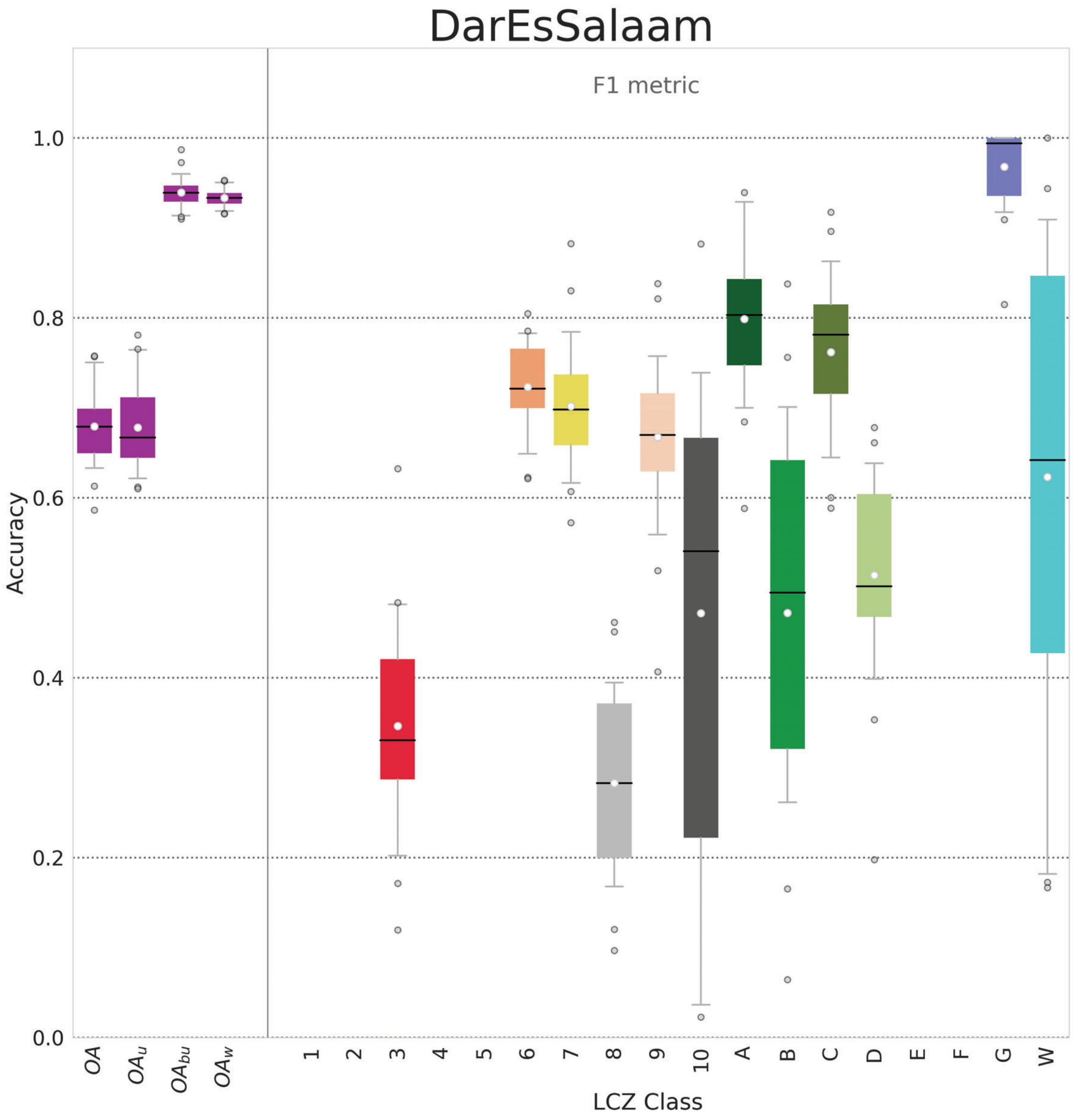

Figure S2d: Same figure as Fig. S2a but for Dar es Salaam

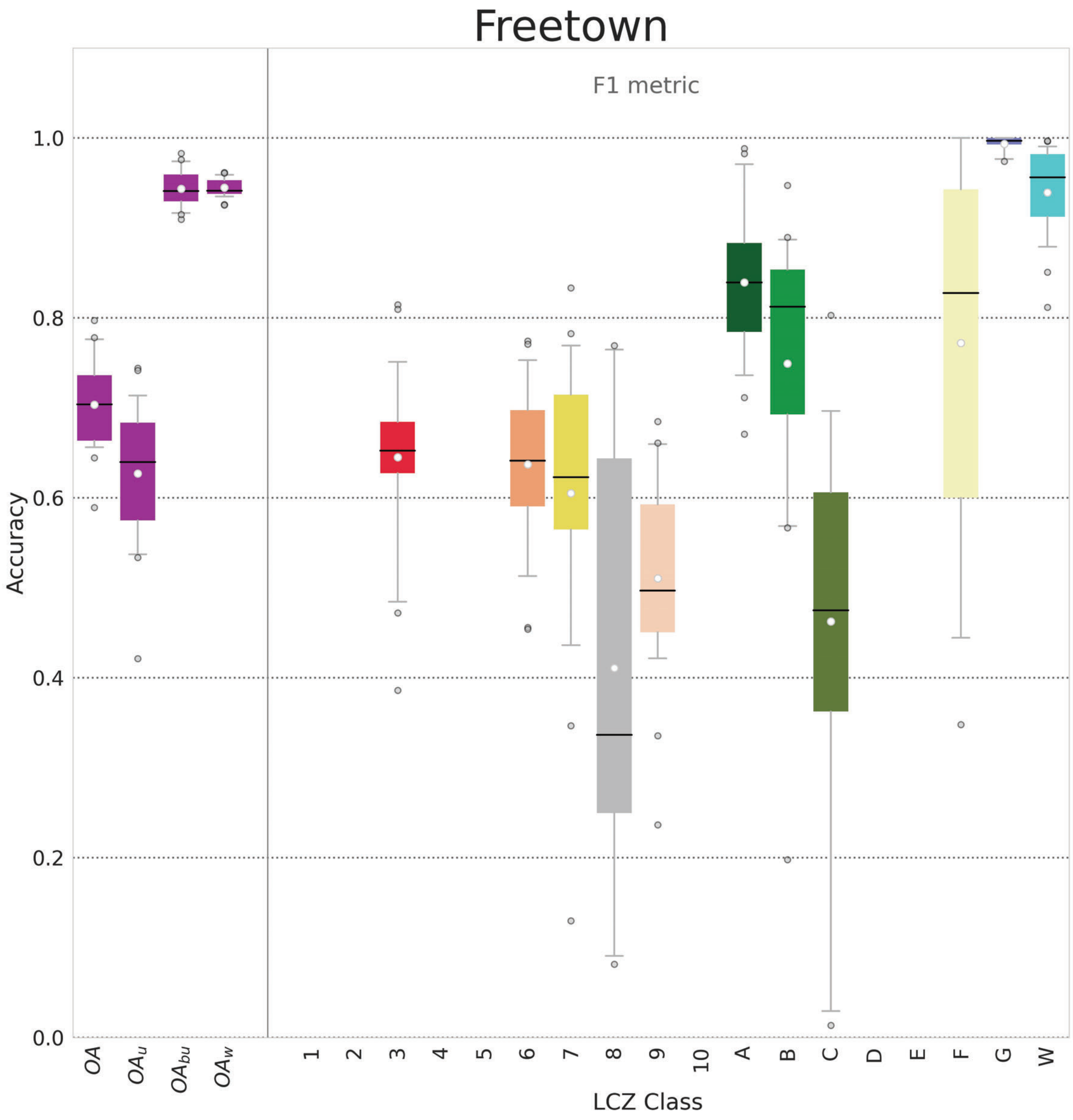

Figure S2e: Same figure as Fig. S2a but for Freetown

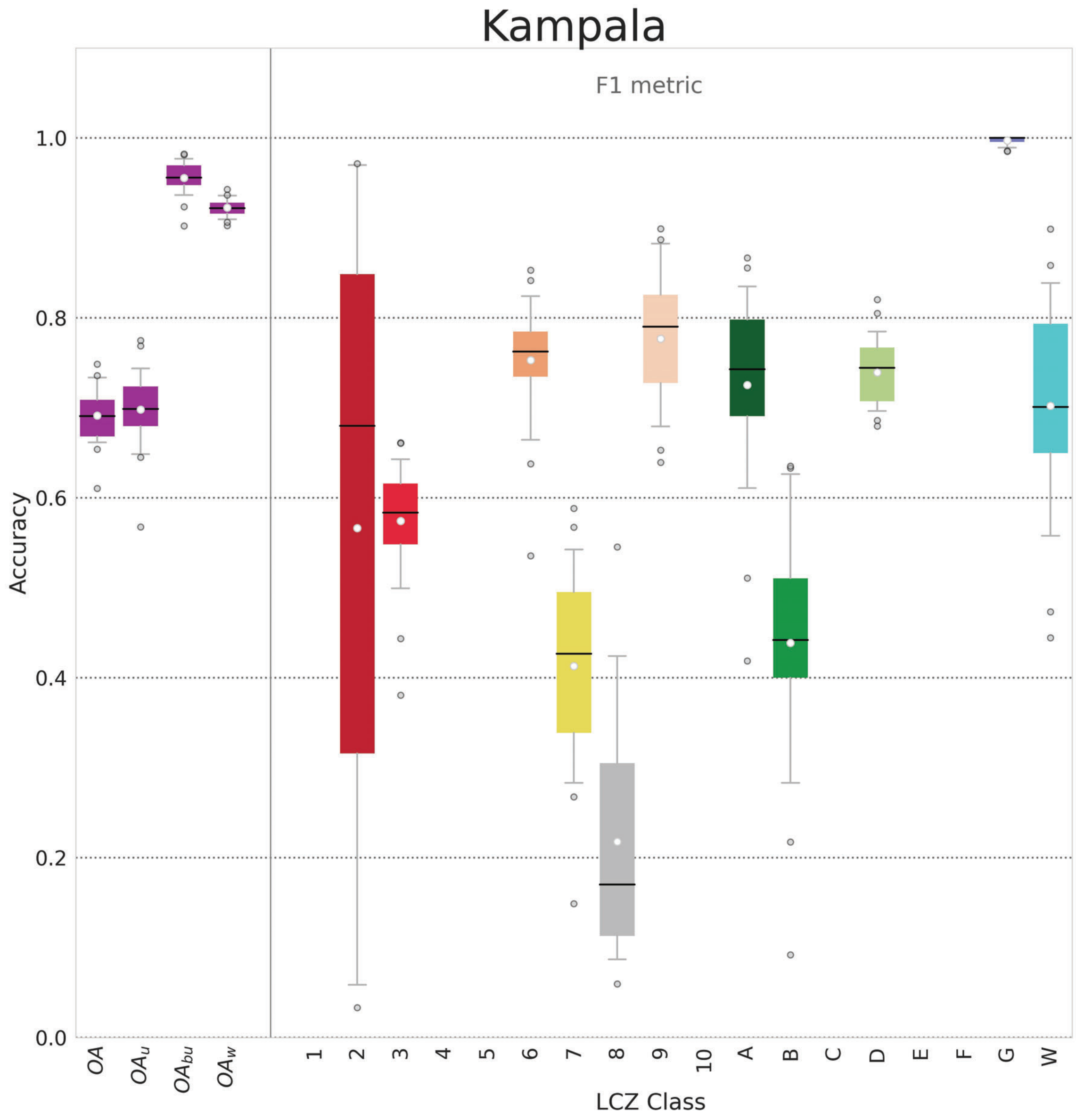

Figure S2f: Same figure as Fig. S2a but for Kampala

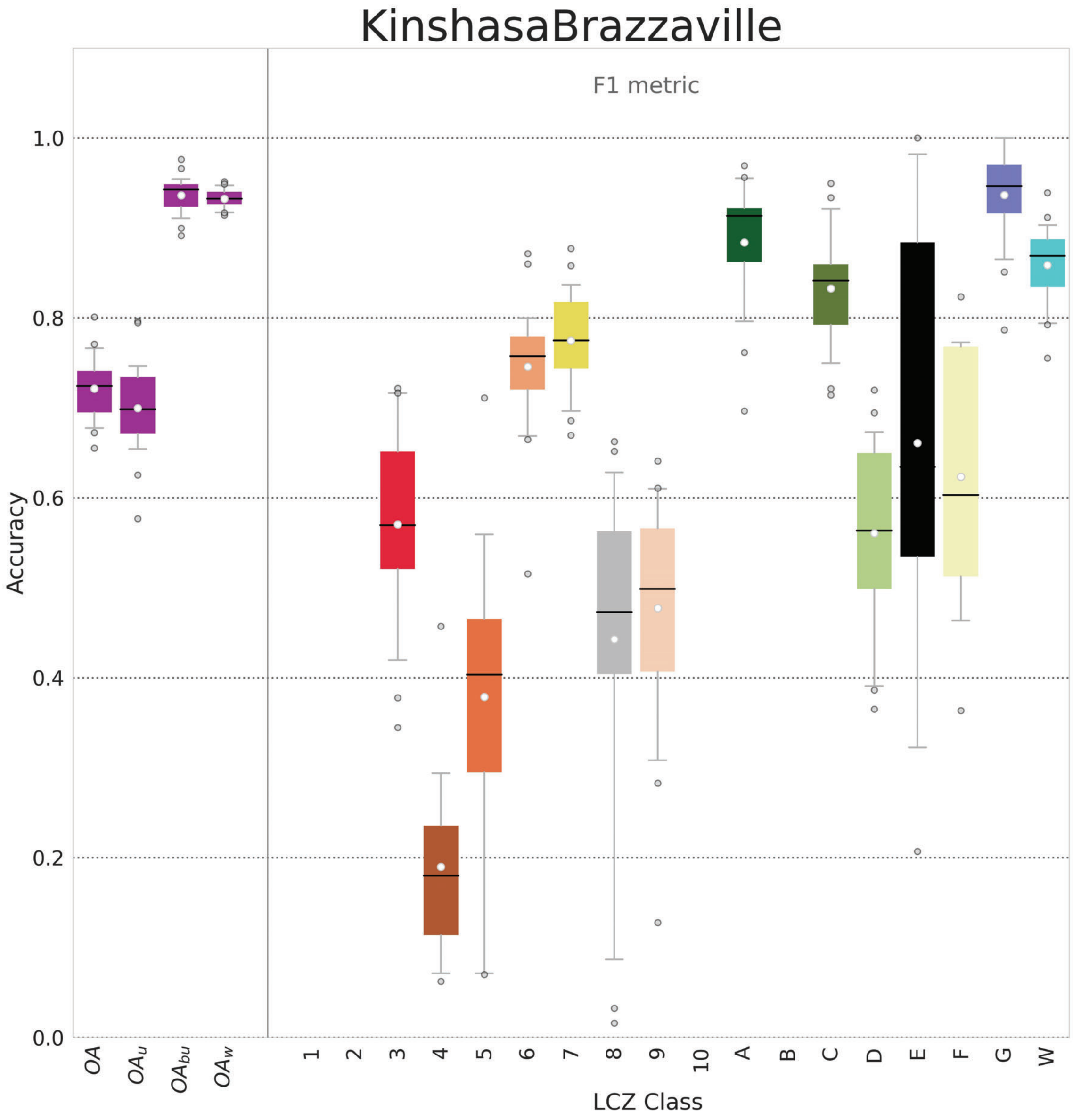

Figure S2g: Same figure as Fig. S2a but for Kinshasa

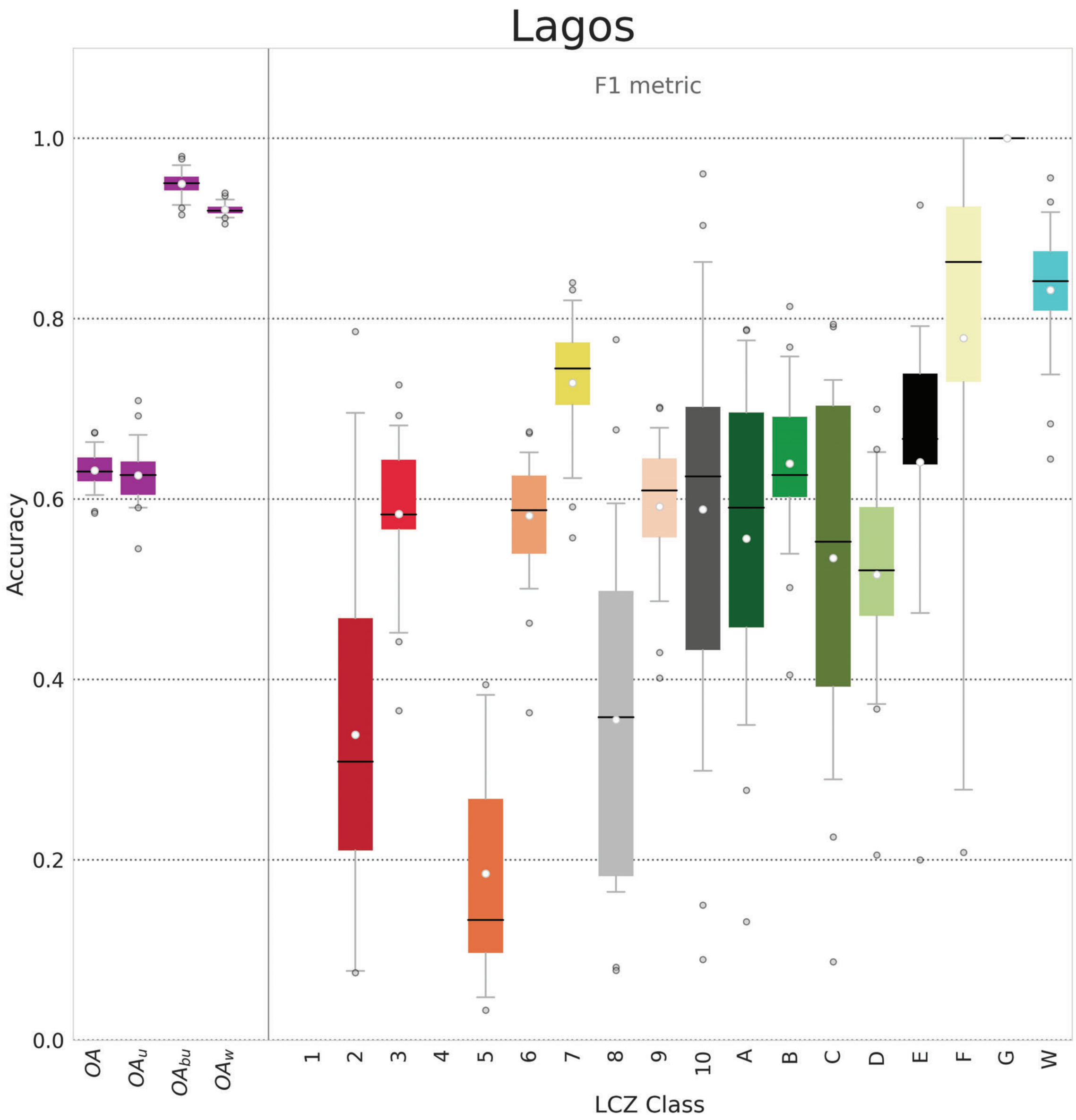

Figure S2h: Same figure as Fig. S2a but for Lagos

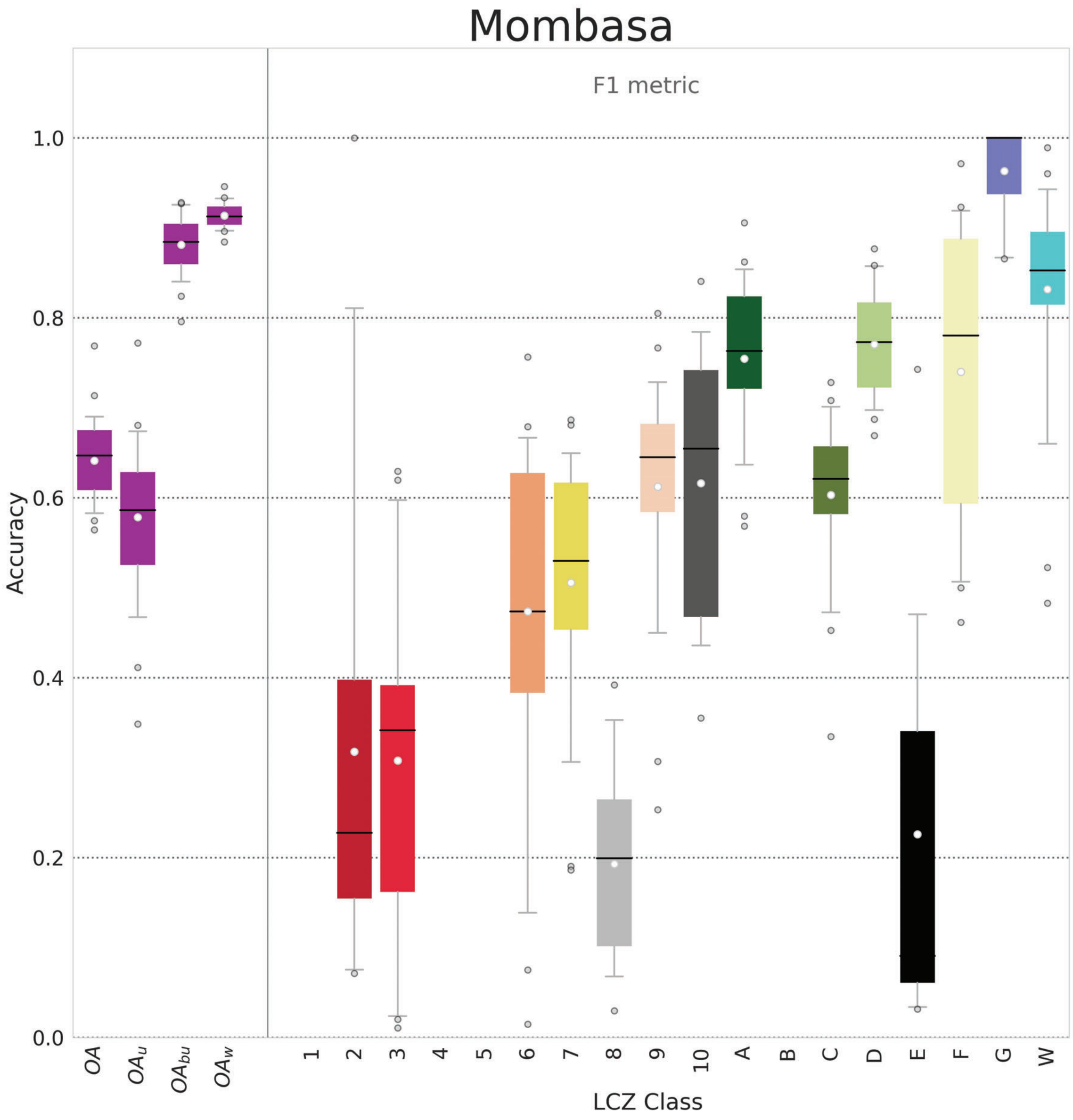

Figure S2h: Same figure as Fig. S2h but for Mombasa

Sensor	Bands, Indices and Ratios	Reference
**Landsat 8**	Median composites for B2 (red), B3 (green), B4 (red), B5 (Near infrared), B6/7 (Shortwave infrared 1/2), B10/11 (Thermal infrared 1/2)	Demuzere *et al.* (2019a)
	Median composites for BCI, NDBaI, EBBI, NDWI, NDBI, NDVI	Demuzere *et al.* (2019a)
	10th and 90th percentile composites for NDVI and BCI	Demuzere *et al.* (2019a)
**Sentinel 1**	Single co-polarization (VV), dual-band cross-polarization (VH) and their ratio (VV/VH)	
	Mean and standard deviation of VV and VH combined	
	VVH indicator	Li *et al.* (2020)
	17 textures from the Gray Level Co-occurrence Matrix (GLCM) (11x11 window size), for both VV and VH: energy, entropy, correlation, inertia, cluster shade, cluster prominence, haralick correlation, mean, variance, dissimilarity, sum average, sum variance, sum entropy, difference of entropies, difference of variances, and two information measures of correlation	Forget *et al.* (2018)
**Sentinel 2**	Median composite Red edge bands (B5, B6, B7)	Forkuor *et al.* (2018)
	Median composite NDVI Red Edge 1 and 2	Forkuor *et al.* (2018)
	Median composite Sentinel-2 Red-Edge Position Index (S2REP)	Kaplan and Avdan (2018)
**Other**	Global Forest Canopy Height (GFCH)	Demuzere *et al.* (2019a)
	DTM, DEM, DSM	Demuzere *et al.* (2019a)

Table S2: Variables list used in the LCZ mapping and respective references that detail their added value. Some of the acronyms given above refer to: Biophysical Composition Index (BCI), Normalized Difference Bareness Index (NDBaI), Enhanced Built-Up and Bareness Index (EBBI), Normalized Difference Wetness Index (NDWI), Normalized Difference Vegetation Index (NDVI), Digital Terrain Model (DTM), Digital Elevation Model (DEM) and Digital Surface Model (DSM).

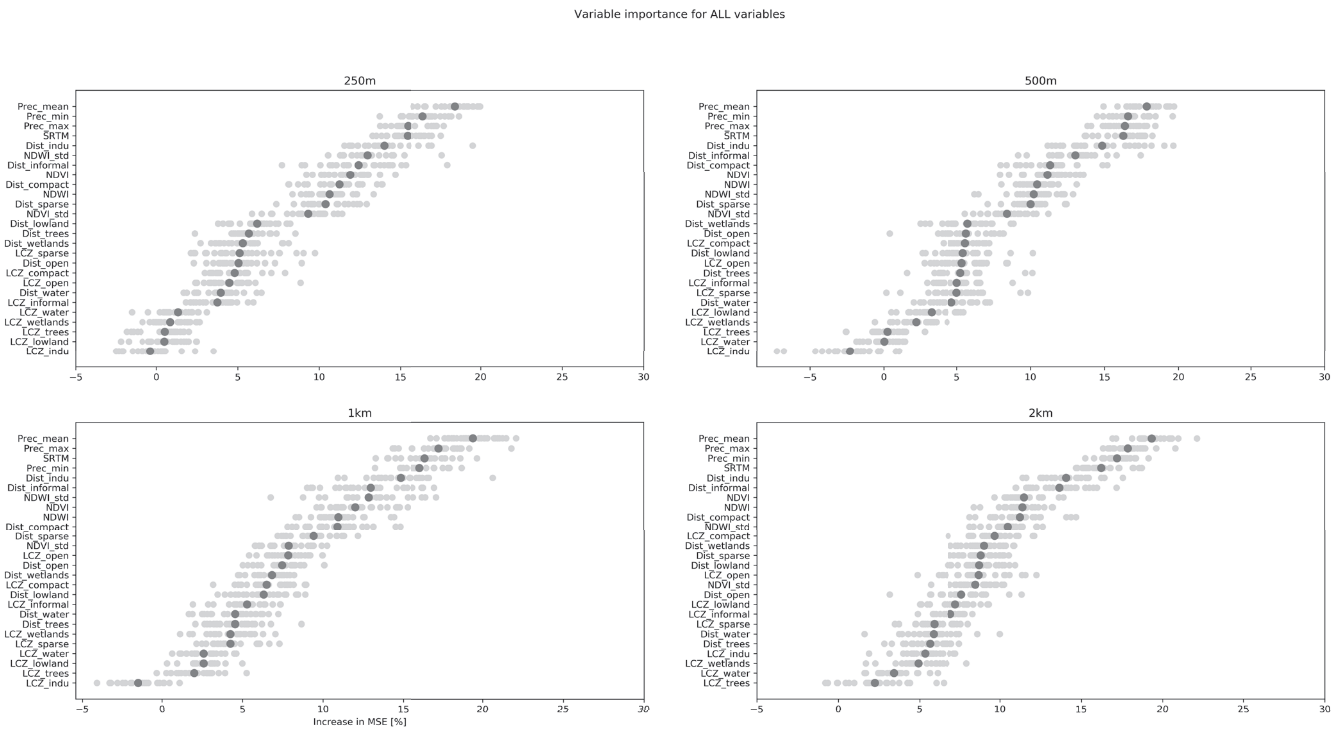

Figure S3: Variable importance by increase in mean square error (%) for all variables (ALL) at each buffer size.
